# Male infertility risk and plasma lipidome: a Mendelian randomization study

**DOI:** 10.3389/fendo.2024.1412684

**Published:** 2024-08-14

**Authors:** Yang Yang, Xinyu Xue, Jun Zhou, Zerui Qiu, Biao Wang, Guangyang Ou, Qing Zhou

**Affiliations:** ^1^ The First Clinical College of Traditional Chinese Medicine, Hunan University of Chinese Medicine, Changsha, China; ^2^ College of Acupuncture & Moxibustion, Tuina, and Rehabilitation, Hunan University of Chinese Medicine, Changsha, China; ^3^ Andrology, The First Hospital of Hunan University of Chinese Medicine, Changsha, China

**Keywords:** male infertility, Mendelian randomization, plasma lipidome, GWAS, sperm quality

## Abstract

**Background:**

In recent years, the decline in sperm quality in men has become a global trend. There is a close relationship between sperm quality and pregnancy outcome. There is a large body of literature supporting the role of plasma lipidome in male infertility, while the complex mechanisms between them and male infertility are still less clear. Systematic study of the causal relationship between plasma lipidome and MI can help to provide new therapeutic ideas and targets for male infertility.

**Methods:**

In this study, we used a two-sample Mendelian randomization analysis based on Genome-wide association studies pooled data of 179 causal relationships between plasma lipidome and male infertility. We used employed the inverse variance weighted method as the main analysis to assess causality between exposure and outcome, in addition to MR-Egger, Weighted median as complementary methods, and tests for multiplicity and heterogeneity.

**Results:**

We identified 13 plasma lipidome comprising 4 types of plasma lipidome that were associated with male infertility. Among these, 9 plasma lipidome were found to be protective factors, while 4 were risk factors. Notably, the largest proportion of these plasma lipidome were triglyceride types, with Sphingomyelin (d40:1) exhibiting the strongest association with male infertility.

**Conclusion:**

These findings contribute to the current better understanding of male infertility and provide new perspectives on the underlying etiology of male infertility as well as prevention and treatment strategies. In addition, clinical trial validation is needed to assess the potential of these plasma lipidome as biomarkers.

## Introduction

Infertility is typically defined as the inability to achieve pregnancy naturally after one year of regular, unprotected sexual intercourse (1).According to the World Health Organization, approximately 50% of infertility cases in couples can be attributed to male factors (2–4). Studies have demonstrated a global decline in male sperm concentration ranging from 1.4% to 1.6% annually, varying by geographical region (5). Exploring the causes of male infertility (MI) and seeking effective treatments remain ongoing scientific pursuits. Single-cell RNA sequencing (sc-RNA-seq) has revealed the molecular complexities of testicular physiology and MI-related diseases. Recent studies have identified key transcriptional profiles of germ cells, linking developmental processes to disease manifestations in the testicular microenvironment (6). These insights provide valuable references for diagnosing and treating unexplained and idiopathic MI. MI has emerged as a significant factor impacting global population dynamics (7), profoundly affecting patients’ psychological well-being, quality of life, family dynamics, and imposing substantial economic and emotional burdens (8). The etiology and pathogenesis of MI remain incompletely understood, with semen abnormalities being a prominent risk factor (1). Lifestyle factors such as smoking (9), alcohol consumption (10), sleep deprivation, obesity, sedentary habits, frequent sexual activity, and adverse psychosocial conditions can all influence semen quality (11, 12). Additionally, obesity (13), sleep deprivation, and lack of physical activity can impact plasma lipidome (PL) levels. Indeed, the lipidome plays a significant role in MI. Previous studies have shown that adipokines, such as adiponectin and chemerin, are closely associated with diseases of the male reproductive system (14). For example, scientific research has demonstrated that adiponectin can mitigate testicular damage in diabetic mice (15). As a result, the lipidome has emerged as a critical area of research. Building on this, we hypothesize whether PL are closely linked to MI as well. In recent years, the study of PL has garnered significant attention. PL serve as crucial energy sources, form the structural and functional basis of biological membranes, and act as essential signaling molecules without which biological processes would not be possible. Modern lipidomics techniques have revolutionized our understanding of the diversity and complexity of circulating lipids. PL encompass a range of lipid classes including cholesteryl ester, ceramide, diacylglycerol, lysophosphatidylcholine, phosphatidylcholine, phosphatidylcholine-ether, phosphatidylethanolamine, phosphatidylethanolamine-ether, sphingomyelin, and triglyceride, among others. The utilization of histological techniques in exploring PL presents a robust platform for comprehensive investigations into the intricate interplay among nutrition, metabolism, and genotypic variability. PL have emerged as significant players strongly linked to human aging (16), cardiometabolic disorders (17), and hematologic disorders (18), offering potential avenues for diagnosis and treatment. However, limited research has delved into their association with MI. Current research has focused on common lipoproteins such as triglycerides (19), high-density lipoprotein, low-density lipoprotein (20), but there is still a lack of comprehensive investigation into the relationship between the complete PL system and MI, warranting further exploration. While the causes of MI have been largely identified by scientists, approximately 40% of patients still have unclear reasons for their condition. Therefore, it is crucial to continue exploring the associated risk factors for its onset (21).

Mendelian randomization (MR) is an emerging epidemiological research approach utilizing genetic variation, particularly single nucleotide polymorphisms (SNPs), as instrumental variables (IVs) for causal inference (22). Randomized controlled trials (RCTs) are the gold standard to establish causal relationships (23). Proper randomization ensures that study groups are comparable in all characteristics, except for the exposure of interest, which often is a therapeutic intervention. However, RCTs cannot always be conducted, because they can be excessively costly, impractical, or even unethical. Mendelian randomization refers to an analytic approach to assess the causality of an observed association between a modifiable exposure or risk factor and a clinically relevant outcome. It presents a valuable tool, especially when randomized controlled trials to examine causality are not feasible and observational studies provide biased associations because of confounding or reverse causality. These issues are addressed by using genetic variants as instrumental variables for the tested exposure: the alleles of this exposure-associated genetic variant are randomly allocated and not subject to reverse causation. This, together with the wide availability of published genetic associations to screen for suitable genetic instrumental variables make Mendelian randomization a time- and cost-efficient approach and contribute to its increasing popularity for assessing and screening for potentially causal associations. An observed association between the genetic instrumental variable and the outcome supports the hypothesis that the exposure in question is causally related to the outcome (24). This method offers advantages in overcoming the limitations of unknown confounders and reverse causality commonly encountered in traditional observational epidemiological studies (25). In our study, we employed a two-sample MR design to investigate the causal relationship between PL and MI. Our aim was to identify potential risk factors and explore possible treatment strategies for MI.

## Methods and materials

### Study design

**Figure 1:**
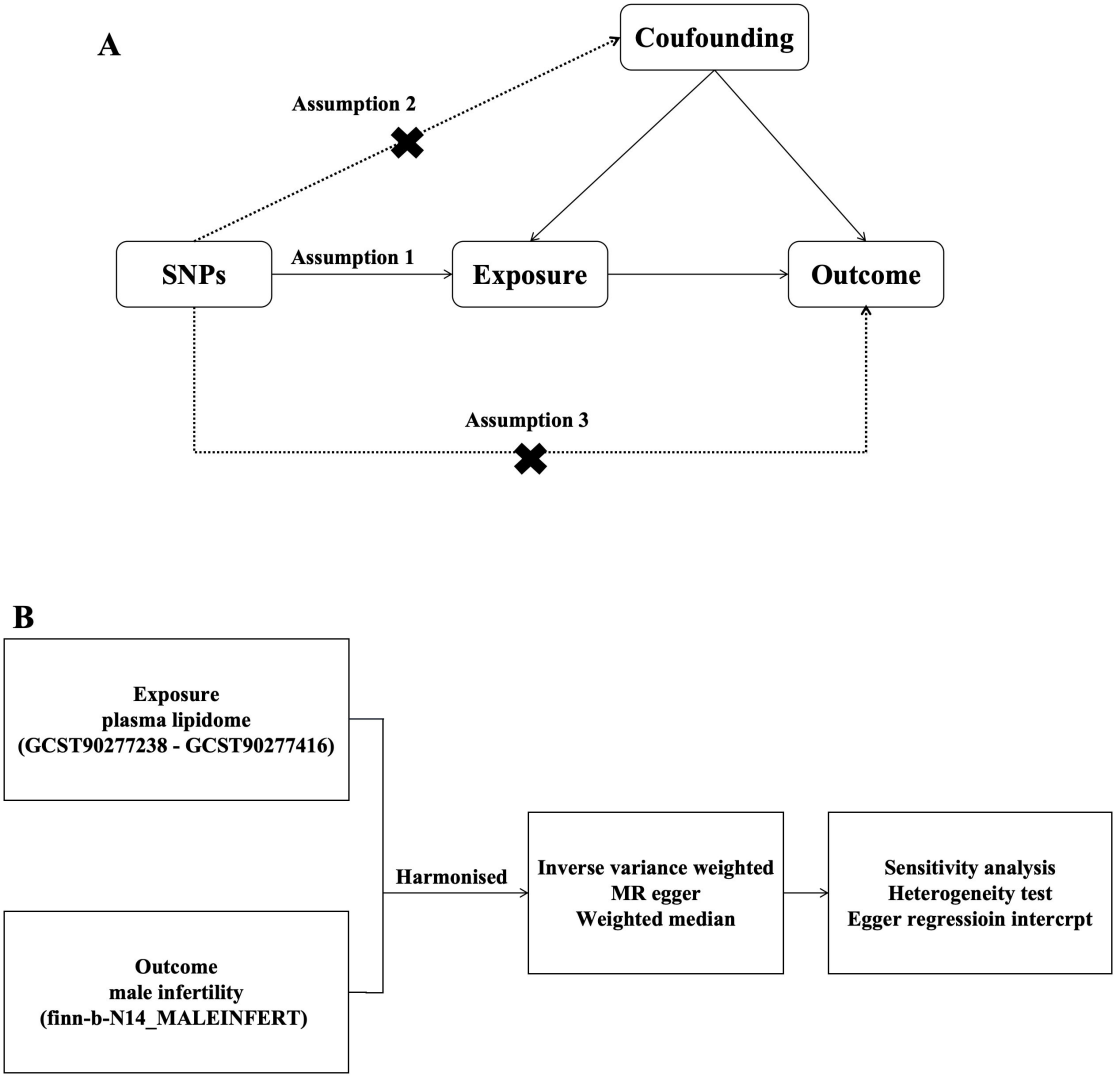
Flow chart. **(A)** Research Flow chart **(B)** Steps of MR analysis.

In this study, PL was selected as the exposure factor, and SNPs associated with PL were selected as IVs. MR of PL and MI was performed using two-sample MR analysis, and heterogeneity and horizontal polytropy were assessed using Cochran’s Q with MR Egger’s method test, and finally sensitivity analyses were performed to verify the stability of the results. We assessed the causal association between 179 PL and MI based on two-sample MR analysis. MR uses genetic variants to represent risk factors; therefore, IVs in causal inference must satisfy three key assumptions MR analysis needs to satisfy 3 conditional assumptions (26): (1) the association assumption: there is a direct correlation between the genetic variants and the exposure factors; (2) Independence assumption: genetic variation is independent of possible confounders between exposure and outcome; (3) Exclusivity assumption: genetic variation can only have an effect on outcome through the pathway of exposure (**Figure 1**).

### Data sources

SNPs significantly associated with PL were selected as IVs, and Genome-wide association studies (GWAS) summary statistics for PL were publicly available from the GWAS catalog (27), which includes 495 genetic associations (GCST90277238 to GCST90277416). The data for MI were downloaded from the IEU Open GWAS database (https://gwas.mrcieu.ac.uk/) under the number finn-b-N14_MALEINFERT dataset, with 8,305 cases in the MI group and 72,799 cases in the normal control group, including 16,377,329 SNPs, and this study population were all European populations.(Year: 2021; Category: Binary; Population: European; ncase: 680; Build: HG19/GRCh37).

### Selection of instrumental variables

We used that exposed SNPs should reach genome-wide significant levels (P < 5 × 10 ^- 8^) as a screening condition (28), and in the absence of significant genome-wide SNPs as IV, SNPs below the genome-wide significance threshold (P < 5 × 10 ^- 6^) were used as a new screening condition (24). to discover more potential causal associations. Meanwhile, to mitigate the bias caused by linkage disequilibrium, r^2^ = 0.001 and kb = 10000 were set as the thresholds for removing linkage disequilibrium (29); only the SNPs with the strongest impact on the outcome were selected as tools. Statistical differences were considered to exist if P < 0. 05, and the F value was set to >10 (29), which indicated the absence of weak instrumental variable bias, calculated as F = R^2^(N-2)/(1-R^2^), where R^2^ is the percentage of variance explained by SNPs in the exposure database and N is the sample size of the exposed GWAS. MI as an outcome was a dichotomous variable and was expressed as odds ratio (OR) and 95% confidence interval (CI). R4.2.3 and R studio software and the R package “Two Sample MR” were used for the above analyses, with a standardized test of α = 0.05. β is the allele effect value and SE is the standard error (SE); finally, the palindromic SNPs were removed by palindromic sequence detection to prevent alleles from influencing the results. Initial studies were obtained with informed consent from all participants, and these data are publicly available on the website. We then used the PhenoScanner V2 website (http://www.phenoscanner.medschl.cam.ac.uk) to exclude SNPs that are potentially confounding factors and related to the outcome (male fertility description) to eliminate the possibility of genetic pleiotropy. After a series of rigorous screenings, the remaining SNPS were considered eligible for IV.

### Mendelian randomization analysis

In this study, several MR methods were used to assess and validate the causal relationship between LP and MI risk, including IVW (30), MR-Egger regression (31), and weighted median (31). IVW is essentially a meta-analysis method that analyzes PL and the effect of MI through weighted linear regression to obtain an overall estimate of the effect of PL and MI. IVW can be used for causal assessment when there is no horizontal pleiotropy between SNPs. If multinomiality exists in the IV, the MR-Egger method shows horizontal multinomiality in the IV by using an intercept term. If the intercept term is equal to 0, the results of MR-Egger regression and IVW are the same. For up to 50% of invalid IVs, the weighted median model yields consistent causal estimation of the relationship.

According to MR, genetic tools can only affect outcomes by exposing people to them, and genetic variants may have pleiotropic effects. In the main analysis, we calculated Wald ratio estimates for each genetic variant and summarized the estimates using an IVW approach. The IVW method with multiplicative random effects provides a parsimonious estimate and takes into account potential heterogeneity among Wald ratio estimates for SNPs. Estimates may be inaccurate if the SNPs used as instruments have horizontal pleiotropic effects that cause results to be influenced by pathways other than exposure. Potential heterogeneity was estimated using the Cochran’s Q test, and fixed-effects IVW models were applied when P > 0.05, when no heterogeneity between SNPs was considered to exist, and random-effects IVW models were applied if P < 0.05, when heterogeneity between SNPs was considered to exist. The sensitivity of the effect estimate after removing SNPs one by one was analyzed by Leave-one-out analysis to assess whether there was an effect of excluding individual SNP observations on the final results. Potential pleiotropy was assessed by intercepts tested by MR-Egger regression, and when P > 0.05, it indicated the absence of pleiotropy.

## Results

### Result of MR

**Figure 2:**
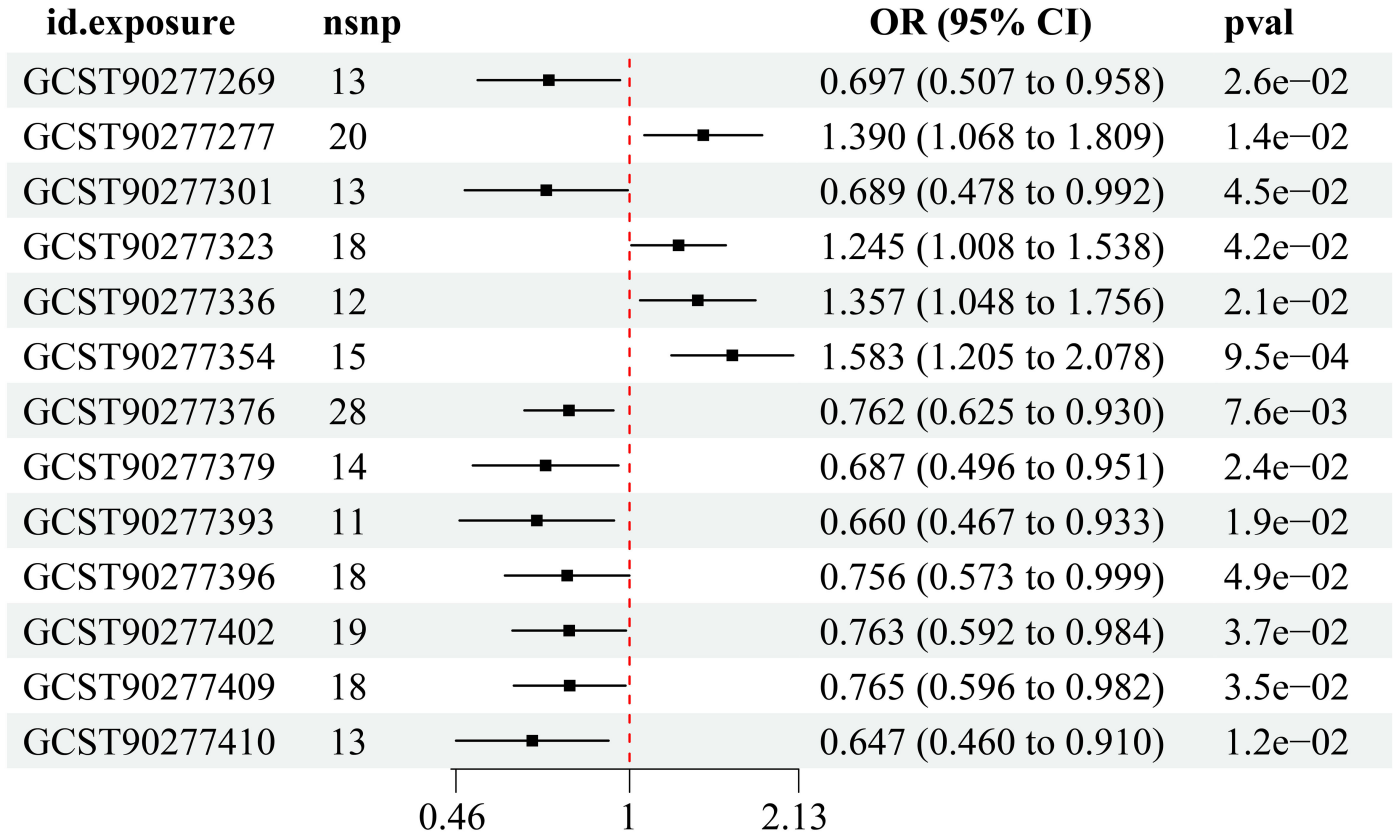
Results of IVW method.

Utilizing instrumental variable screening principles, a total of 13 PL were identified as causally associated with MI by at least one MR method (p < 0.05): four were identified as risk factors, while nine were deemed protective factors (**Figure 2**).

IVW analysis showed (**Table 1**) that Phosphatidylethanolamine (18:0_0:0) levels [OR = 0.697, 95% CI (0.507 - 0.958), p = 0.026], Phosphatidylcholine (18:0_18:3) levels [OR = 0.689, 95% CI (0.478 - 0.992), p = 0.045], Sphingomyelin (d40:1) levels [OR = 0.762, 95% CI (0.625 - 0.930), p = 0.0076], Triacylglycerol (46:1) levels [OR = 0.687, 95% CI (0.496 - 0.951), p = 0.024], Triacylglycerol (51:2) levels [OR = 0.660, 95% CI (0.467 - 0.933), p = 0.019], Triacylglycerol (52:2) levels [OR = 0.756, 95% CI (0.573 - 0.999), p = 0.049], Triacylglycerol (53:3) levels [OR = 0.763, 95% CI (0.592 - 0.984), p = 0.037], Triacylglycerol (56:3) levels [OR = 0.765, 95% CI (0.596 - 0.982), p = 0.035], Triacylglycerol (56:4) levels [OR = 0.647, 95% CI (0.460 - 0.910), p = 0.012] were protective factors for MI. Phosphatidylcholine (16:0_16:0) levels [OR = 1.390, 95% CI (1.068 - 1.809), p = 0.014], Phosphatidylcholine (O-16:0_20:4) levels [OR = 1.245, 95% CI (1.008 - 1.538), p = 0.042], Phosphatidylcholine (O-18:0_20:4) levels [OR = 1.357, 95% CI (1.048 - 1.756), p = 0.021], Phosphatidylethanolamine (O-18:1_20:4) levels [OR = 1.583, 95% CI (1.205 - 2.078), p = 0.001] risk of MI was a risk factor.

**Table 1:** Results of two-sample MR.

Exposure	SNP	MR method	OR	95%LCI	95%UCI	p-value
Phosphatidylethanolamine (18:0_0:0) levels	13	IVW	0.697	0.507	0.958	0.026
		Weighted median	0.640	0.423	0.966	0.034
		MR Egger	0.430	0.166	1.113	0.110
Phosphatidylcholine (16:0_16:0) levels	20	IVW	1.390	1.068	1.809	0.014
		Weighted median	1.485	1.023	2.157	0.038
		MR Egger	1.540	0.713	3.329	0.286
Phosphatidylcholine (18:0_18:3) levels	13	IVW	0.689	0.478	0.992	0.045
		Weighted median	0.864	0.529	1.410	0.558
		MR Egger	0.344	0.147	0.806	0.032
Phosphatidylcholine (O-16:0_20:4) levels	18	IVW	1.245	1.008	1.538	0.042
		Weighted median	1.202	0.911	1.584	0.193
		MR Egger	1.139	0.788	1.647	0.499
Phosphatidylcholine (O-18:0_20:4) levels	12	IVW	1.357	1.048	1.756	0.021
		Weighted median	1.302	0.950	1.786	0.101
		MR Egger	1.423	0.842	2.406	0.217
Phosphatidylethanolamine (O-18:1_20:4) levels	15	IVW	1.583	1.205	2.078	0.001
		Weighted median	1.403	0.965	2.039	0.076
		MR Egger	1.137	0.588	2.201	0.709
Sphingomyelin(d40:1)levels	28	IVW	0.762	0.625	0.930	0.008
		Weighted median	0.623	0.462	0.841	0.002
		MR Egger	0.589	0.420	0.828	0.005
Triacylglycerol (46:1) levels	14	IVW	0.687	0.496	0.951	0.024
		Weighted median	0.765	0.492	1.190	0.235
		MR Egger	0.429	0.183	1.008	0.076
Triacylglycerol (51:2) levels	11	IVW	0.660	0.467	0.933	0.019
		Weighted median	0.662	0.410	1.069	0.091
		MR Egger	0.680	0.285	1.621	0.406
Triacylglycerol (52:2) levels	18	IVW	0.756	0.573	0.999	0.049
		Weighted median	0.732	0.486	1.103	0.135
		MR Egger	0.949	0.491	1.836	0.879
Triacylglycerol (53:3) levels	19	IVW	0.763	0.592	0.984	0.037
		Weighted median	0.743	0.508	1.087	0.126
		MR Egger	0.586	0.306	1.123	0.125
Triacylglycerol (56:3) levels	18	IVW	0.765	0.596	0.982	0.035
		Weighted median	0.698	0.491	0.991	0.044
		MR Egger	0.691	0.364	1.310	0.274
Triacylglycerol (56:4) levels	13	IVW	0.647	0.460	0.910	0.012
		Weighted median	0.592	0.398	0.882	0.010
		MR Egger	0.832	0.251	2.755	0.769

MI, male infertility; MR, Mendelian randomization; IVW, Inverse variance weighted; SNP, single nucleotide polymorphism; LCI, lower confidence interval; UCI, upper confidence interval.

**Figure 3:**
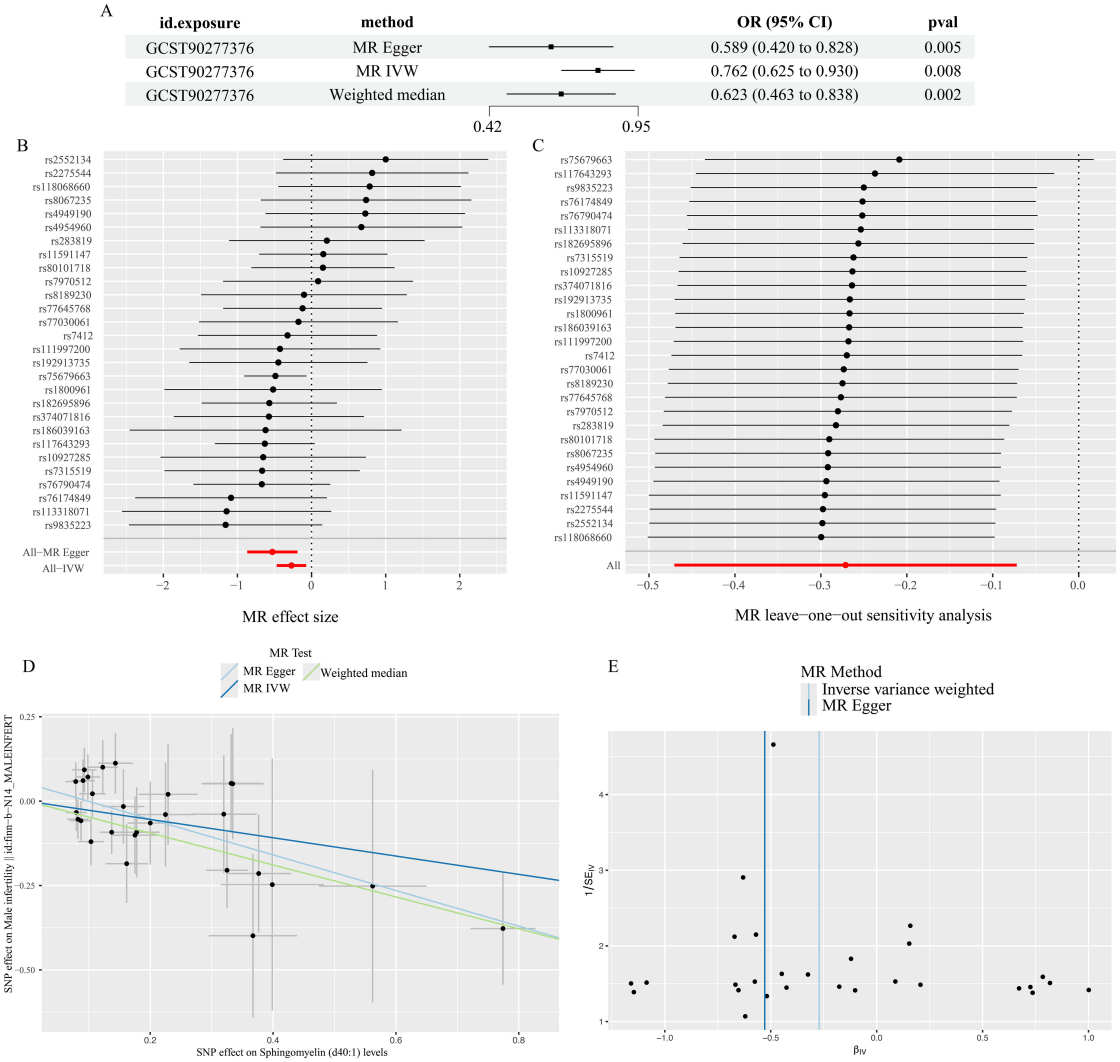
Results of Sphingomyelin (d40:1) levels analysis. **(A)** results of three methods **(B)** forest plot **(C)**leave-one-out plot **(D)**scatter plot **(E)**funnel plot.

For Sphingomyelin (d40:1) levels, the p-value of all three methods (IVW, weighted median, and MR-Egger) was less than 0.05 (**Figure 3**), and we therefore concluded that Sphingomyelin (d40:1) levels are the PL most closely associated with MI.

### Heterogeneity test, sensitivity analysis, multiple validity analysis

The results of Cochran’s Q and MR-Egger regression methods showed that there was no significant heterogeneity or pleiotropy in this study. The results of leave-one-out method showed that the results did not change after removing SNPs one by one. These analyses proved to some extent the robustness of the results of this study (**Table 2**).

**Table 2:** Heterogeneity tests and Test for directional horizontal pleiotropy.

Id.exposure	Exposure	Heterogeneity tests		Test for directional horizontal pleiotropy	
		IVW Q_pvalue	MR Egger Q_pvalue	Egger_intercept	Egger_intercept pvalue
GCST90277269	Phosphatidylethanolamine (18:0_0:0) levels	0.720	0.741	0.066	0.314
GCST90277277	Phosphatidylcholine (16:0_16:0) levels	0.782	0.732	-0.014	0.784
GCST90277301	Phosphatidylcholine (18:0_18:3) levels	0.375	0.545	0.087	0.107
GCST90277323	Phosphatidylcholine (O-16:0_20:4) levels	0.762	0.725	0.018	0.573
GCST90277336	Phosphatidylcholine (O-18:0_20:4) levels	0.725	0.646	-0.010	0.841
GCST90277354	Phosphatidylethanolamine (O-18:1_20:4) levels	0.663	0.683	0.053	0.301
GCST90277376	Sphingomyelin(d40:1) levels	0.488	0.621	0.052	0.078
GCST90277379	Triacylglycerol (46:1) levels	0.653	0.692	0.072	0.265
GCST90277393	Triacylglycerol (51:2) levels	0.825	0.752	-0.005	0.945
GCST90277396	Triacylglycerol (52:2) levels	0.449	0.418	-0.038	0.466
GCST90277402	Triacylglycerol (53:3) levels	0.609	0.594	0.039	0.398
GCST90277409	Triacylglycerol (56:3) levels	0.540	0.477	0.017	0.740
GCST90277410	Triacylglycerol (56:4) levels	0.159	0.124	-0.034	0.675

MR, Mendelian randomization; IVW, Inverse variance weighted.

## Discussion

With the accelerated pace of life and increased work pressure, MI has become a social problem of general concern (32). MI is a global health problem that requires attention from the medical profession. By pooling data from 16 studies (33), researchers found that Central and Eastern Europe had the highest prevalence of MI (8-12%). MI leads to serious adverse health, psychological, social, and economic consequences related to the disease. Results have shown that infertile men have a higher risk of cancer (e.g., testicular cancer, prostate cancer) and other adverse health outcomes (e.g., heart disease, diabetes, and autoimmune disorders) than the normally fertile population (34–36), so it is important to characterize the etiology of MI and treat the symptoms.

MR analysis is a scientific and effective method to identify the causal relationship between risk factors and diseases.MR is an effective scientific method for identifying causal relationships between risk factors and diseases. It can be used to evaluate the causality between modifiable exposures or risk factors and clinically relevant outcomes. MR uses genetic variations as instrumental variables, which are randomly assigned. Compared to observational studies, this method reduces the influence of confounding factors. Moreover, since genetic variations are determined at birth and are not influenced by outcomes, it can avoid the interference of reverse causation. The wide availability of published genetic associations for screening suitable genetic instrumental variables makes Mendelian randomization a time - and cost - efficient approach, contributing to its increasing popularity for assessing and screening potentially causal associations. Scientists have confirmed numerous mechanisms affecting male infertility, yet many unknown areas still warrant exploration by researchers. In the context of declining global sperm quality, it is crucial to employ scientific research and experimental methods to explore factors influencing MI and develop new treatments. MR is a robust method for identifying causal relationships, and many researchers are actively using this approach to investigate various factors affecting male infertility (37, 38). Although MR cannot replace RCTs, it can provide supplementary evidence or offer new design insights for RCTs, thereby enhancing clinical diagnosis and treatment strategies. Thus, utilizing MR to uncover causal relationships related to MI is highly important. In this study, we employed MR to comprehensively assess the impact of 495 PL on MI. Our MR results indicate that PL may significantly influence MI, suggesting that lipoproteins play a crucial role in its pathogenesis.

PL refer to the overall composition and characterization of all lipid molecules in plasma. Lipids are a class of biomolecules that play important roles in maintaining cell membrane structure, energy storage, and signaling. The analysis of PL can help us to understand the lipid metabolism of an individual or a group of individuals in healthy and diseased states, which is important for the study of metabolic diseases, reproductive health and so on. There is a large body of literature supporting that PL play an important role in MI (39), and PL has emerged as a comprehensive approach to identify specific biomarkers associated with reproductive disorders (40, 41).Researchers have noted that low sperm counts in men are associated with abnormal PL levels and have suggested that fertility assessment could be improved by increasing lipid screening (42). And abnormalities in PL may directly affect sperm formation and function (43).

Through our study we found a total of 13 PL associated with MI in the European population, which were Phosphatidylethanolamine(18:0_0:0) levels, Phosphatidylcholine (18:0_18:3) level, Phosphatidylcholine (18:0_18:3) levels, Phosphatidylcholine (O-16:0_20:4) levels, Phosphatidylcholine (O-18:0_20:4) levels, Phosphatidylethanolamine (O-18:1_20:4) levels, Sphingomyelin(d40:1)levels, Triacylglycerol (46:1) levels, Triacylglycerol (51:2) levels, Triacylglycerol (52:2) levels, Triacylglycerol (53:3) levels, Triacylglycerol (56:3) levels, Triacylglycerol (56:4) levels。The most diverse of these were triglycerides, and all six were protective factors. Triglycerides are important determinants of sperm composition. It has been suggested that special attention should be paid to triglycerides in classical lipid screening because it may be a sensitive indicator of male reproductive dysfunction (19), which has a consistent outlook with our findings. Many studies have found that sperm immaturity may be and male reproductive defects with dyslipidemia, and a relevant meta-analysis showed that triglycerides do have a significant correlation with semen parameters (44).Correnti et al. conducted a multilevel analysis of PL in 50 subjects (31 infertile patients and 19 normal fertile men) (45). Triglyceride (14:0_16:0_18:0) showed a positive correlation with MI, while negative correlations were observed for phosphatidylcholine (PC O-16:2_18:1)-CH3, phosphatidylethanolamine (PE O-16:1_20:3), etc., which are consistent with our findings. However, some researchers have also suggested (46) a significant negative correlation between riglycerides and semen parameters as well as serum total testosterone. For instance, Alterman et al. suggested (47) that lipid parameters such as triglycerides are negatively correlated with sperm morphology. Conversely, some scientists argue that lipid levels do not significantly correlate with MI. This discrepancy conflicts with our results, highlighting the dual nature of triglycerides. Lipid testing is commonly accessible in hospitals. Historically, elevated triglycerides have been a primary concern, and we advocate for maintaining triglyceride levels within reasonable limits, neither too high nor too low. Timely intervention is recommended in cases where triglyceride levels are excessively low.

In addition, we also discovered that among the 13 relevant PL, Sphingomyelin (d40:1) levels exhibited the strongest association with MI. Experimental evidence has demonstrated that men with oligospermia exhibit a significant reduction in sphingomyelin levels compared to normal men, indicating its protective role (48), which is consistent with our findings. Sphingomyelin is the most abundant phospholipid in spermatozoa and plays a crucial role in sperm maturation, being an essential component of the epididymis (49). In various types of cells, different stimuli can activate sphingomyelinase to hydrolyze sphingolipids. Sphingomyelin plays a crucial role in the hydrolysis of sperm phospholipids, which is closely linked to sperm capacitation and fertilization processes. Additionally, it can inhibit gonadotropin-induced testosterone synthesis in mesenchymal cells and reduce gonadotropin binding to receptors. This not only decreases androgen synthesis but also leads to excessive apoptosis of spermatogonia and spermatocytes (50). Wittmann et al. (51) conducted an experimental study and found that sphingomyelinase 1 is indispensable for fertility in mice. Similarly, Datar (52) discovered that metabolites of sphingomyelin, triglycerides, etc., are closely associated with testicular growth in mice.

Sphingolipids, a family of lipids with a common sphingomyelin backbone, are integral to various physiological and pathological processes in cells. They act as important regulators of cellular processes such as cell differentiation and apoptosis, and are universally expressed in all mammalian cells. During epididymal transit, there occurs an exchange of sphingolipids between spermatozoa and the surrounding fluid. Sphingolipid metabolites in the reproductive system have garnered significant attention in recent years. Studies have indicated (53) a potential association between sphingolipids and impaired gonadal function as well as infertility. In our study, sphingomyelin emerged as a protective factor for MI. This leads us to consider focusing on the measurement and preservation of sphingomyelin levels in future clinical treatments, particularly for patients with unexplained MI. This approach offers new perspectives for clinical management strategies.

Generally, men with primary infertility tend to have poorer health compared to fertile men, necessitating more reliable diagnostic tools and robust personalized interventions. Through our study, we identified 13 PL as major contributors to MI. We advocate for a more comprehensive assessment of PL in men seeking fertility, incorporating it as a routine part of clinical evaluations for infertility, beyond the standard PL tests. Special attention should be given to the screening of sphingomyelins and triglycerides, thereby enhancing the diagnostic tools for MI. Diet (40) and obesity (17) are recognized as risk factors for MI. Therefore, it is crucial to conduct thorough PL screening, particularly in men with higher BMI or obesity. Even in young patients without overt lipid abnormalities (such as severe overweight, obesity, and metabolic syndrome), PL levels should be meticulously monitored. Early intervention for those with abnormal PL levels can mitigate the adverse effects on fertility. In the realm of pharmacological research for MI, regulating PL levels, especially sphingomyelins and triglycerides, could be a promising approach. Developing new targeted drugs that modulate various PL offers potential new treatment options for MI. Additionally, clinical education should emphasize the impact of diet and obesity on fertility. From what we know, this study stands as the inaugural in-depth investigation into the causal connection between all categories of PL and MI. Our study design was meticulously crafted to minimize confounding factors and potential sources of observational bias. All instrumental variables used were sourced from publicly available GWAS, ensuring a rich dataset and statistical robustness in assessing the plasma lipoproteins relevant to male fertility.

In the future, we deem it necessary to continue conducting RCTs to ascertain the precise role of PL in MI. Our study findings provide a robust theoretical foundation for similar RCTs. Furthermore, our research has identified PL as both protective factors and risk factors for MI, guiding early interventions, treatments, and prognostic assessments. Future large-scale studies and RCTs on PL will further elucidate the causal relationships between these PL and MI. Promoting PL screening globally can provide new strategies and methods to improve male fertility.

## Limitation

Our study has several limitations. Firstly, the results of the analysis are specific to European populations and may not accurately reflect populations in other regions. Secondly, there is potential bias in the study due to the relatively small number of MI cases in the GWAS data. Lastly, the GWAS for MI was not categorized, which limits our ability to provide detailed insights into the association of PL with specific types of MI. Future studies and randomized controlled trials are necessary to elucidate the causal relationship between PL and MI more effectively.

## Conclusion

MR analyses using large datasets analyzed by GWAS have revealed a causal relationship between PL and MI. However, a larger GWAS database is necessary to further investigate the mechanisms underlying this association. Sphingomyelin and triglycerides are closely linked to the development of MI, and effective control of these lipid levels can potentially reduce the prevalence of MI, providing new avenues for treatment and prevention.

## Data Availability

The original contributions presented in the study are included in the article/**Supplementary Material**, further inquiries can be directed to the corresponding author.

## Supplementary material

Supplementary Table 1 Characteristics of SNPs used as instrumental variables for Sphingomyelin(d40:1) levels.
